# Enhanced Inhibition of Tumorigenesis Using Combinations of miRNA-Targeted Therapeutics

**DOI:** 10.3389/fphar.2019.00488

**Published:** 2019-05-16

**Authors:** Svetlana Miroshnichenko, Olga Patutina

**Affiliations:** Laboratory of Nucleic Acids Biochemistry, Institute of Chemical Biology and Fundamental Medicine SB RAS, Novosibirsk, Russia

**Keywords:** oncogenic miRNA, cancer, antisense oligonucleotide, miRNA mimic, chemotherapy, temozolomide, gemcitabine

## Abstract

The search for effective strategies to inhibit tumorigenesis remains one of the most relevant scientific challenges. Among the most promising approaches is the direct modulation of the function of short non-coding RNAs, particularly miRNAs. These molecules are propitious targets for anticancer therapy, since they perform key regulatory roles in a variety of signaling cascades related to cell proliferation, apoptosis, migration, and invasion. The development of pathological states is often associated with deregulation of miRNA expression. The present review describes in detail the strategies aimed at modulating miRNA activity that invoke antisense oligonucleotide construction, such as small RNA zippers, miRNases (miRNA-targeted artificial ribonucleases), miRNA sponges, miRNA masks, anti-miRNA oligonucleotides, and synthetic miRNA mimics. The broad impact of developed miRNA-based therapeutics on the various events of tumorigenesis is also discussed. Above all, the focus of this review is to evaluate the results of the combined application of different miRNA-based agents and chemotherapeutic drugs for the inhibition of tumor development. Many studies indicate a considerable increase in the efficacy of anticancer therapy as a result of additive or synergistic effects of simultaneously applied therapies. Different drug combinations, such as a cocktail of antisense oligonucleotides or multipotent miRNA sponges directed at several oncogenic microRNAs belonging to the same/different miRNA families, a mixture of anti-miRNA oligonucleotides and cytostatic drugs, and a combination of synthetic miRNA mimics, have a more complex and profound effect on the various events of tumorigenesis as compared with treatment with a single miRNA-based agent or chemotherapeutic drug. These data provide strong evidence that the simultaneous application of several distinct strategies aimed at suppressing different cellular processes linked to tumorigenesis is a promising approach for cancer therapy.

## Introduction

Tumorigenesis represents a complex process characterized by several hallmarks including fast, uncontrolled cell proliferation, reprogramming of energy metabolism, resistance to cell death and replicative senescence, immortalization, evasion of immune surveillance, abundant vascularization, infiltrated growth, and metastasis (Markopoulos et al., 2017). Each of these events is regulated by numerous non-coding molecules, such as long non-coding RNAs and small non-coding RNAs, in particular miRNAs (Zheng et al., 2017). MiRNAs appear to be the key markers of pathological conditions and significant therapeutic targets, since tumorigenesis is commonly associated with aberrant miRNA expression and an imbalance between tumor suppressor and oncogenic miRNAs.

The present review mainly encompasses publications from 2015 to 2018, and contains very recent data on miRNA turnover and function in normal tissues and during pathological states, as well as the latest achievements in the development of sequence-specific approaches for regulating miRNA function and activity. The main informative load of the review focuses on a comparative analysis of the antitumor efficiency of *in vitro* and *in vivo* application of various combinations of current miRNA-based therapeutics, in addition to their efficacy in conjunction with chemotherapy. The most promising combination schemes that exhibit additive or synergistic influence on tumor growth *in vitro* and *in vivo* are illuminated in the present paper.

## Different Scenarios of miRna Biogenesis

**FIGURE 1:**
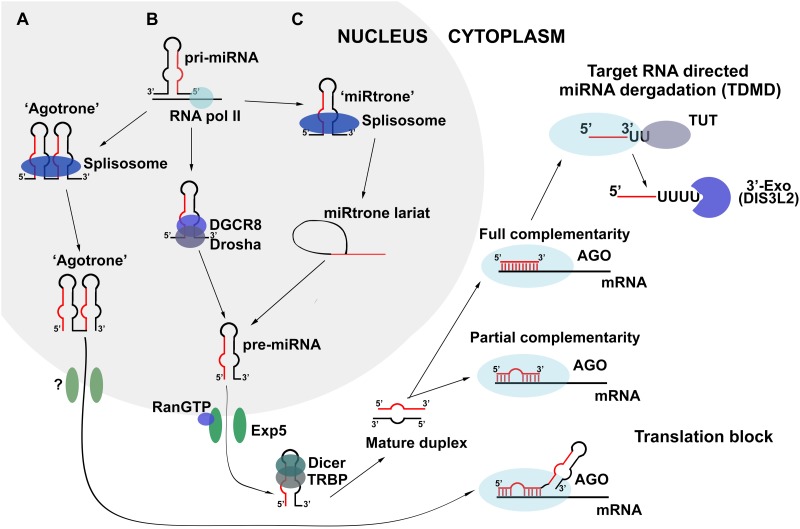
Biogenesis and turnover of miRNA in the cell. **(A,C)** Non-canonical schemes of biogenesis: Drosha/Dicer-independent and Drosha-independent, respectively; **(B)** Canonical Drosha/Dicer-dependent scheme of miRNA biogenesis.

miRNA biogenesis is a complicated process accomplished by several enzyme complexes. In contrast to exogenously created siRNAs, miRNAs are generated endogenously by RNA polymerase II from protein-coding sequences or individual miRNA promoters (Lee et al., 2004; Figure 1). The produced transcript is called pri-miRNA and comprises a set of miRNA copies encoded in hairpin structures. Each hairpin, consisting of a 33-35 bp stem and terminal loop, is recognized and specifically processed by the Drosha/DGCR8 enzyme complex (Figure 1B). Drosha/DGCR8 forms short hairpin RNA with a 2-nt overhang at the 3′-end called pre-miRNA, which is further transported from the nucleus by the protein complex, EXP5/Ran, and disposed of in the cytoplasm using GTP hydrolysis as an energy source. Further transformations of pre-miRNA mediated by the Dicer enzyme include recognition of the 3′ overhang, excision of the loop from the hairpin structure, and formation of a linear miRNA duplex (Figure 1). Such a duplex consists of two strands differentiated by their stability; a guide strand representing mature miRNA that performs the regulatory function and a passenger strand that is usually degraded by intracellular exonucleases. During the final step of miRNA biogenesis, proteins belonging the Ago family conduct distinction of the strands, duplex melting, and initiation of passenger strand degradation. Moreover, Ago proteins not only provide conclusive maturation of miRNA but also control miRNA activity as regulators of gene expression (Daugaard and Hansen, 2017). Such a canonical scheme of biogenesis is typical for the majority of intracellular miRNAs; however, some molecules are known to be formed by alternative pathways, which are characterized by the absence of one or several processing steps.

In Dicer-independent biogenesis, canonically formed pre-miRNA escapes Dicer processing and interacts directly with the Ago2 protein following export to the cytoplasm (Daugaard and Hansen, 2017). Since Ago2 is not able to catalyze the further maturation of pre-miRNA due to its hairpin structure, it induces a single-stranded break in the hairpin region and initiates the exonuclease degradation of the passenger strand of pre-miRNA, which finishes with the formation of conventional mature miRNA. Another alternative avenue for miRNA maturation is Drosha/DGCR8-independent biogenesis (Figure 1C). In such a scheme, the pri-miRNA molecule represents copies of sequences called “miRtron” that comprise the acceptor and donor sites for splicing. The splicing of pri-miRNA replaces the processing by Drosha/DGCR8 and promotes the formation of pre-miRNA, further maturation of which occurs according to the canonical scheme of biogenesis (Daugaard and Hansen, 2017; Figure 1C).

There exists another biogenesis pathway that does not depend on the action of either Drosha/DGCR8 or Dicer complexes, and can be realized in the presence of “agotron” sequences in the genome (Figure 1A). Being subjected to splicing, “agotron” represents an analog of “miRtron” that binds to Ago proteins following transcription and translocation to the cytoplasm. “Agotron” regulates gene expression in the same manner as miRNA, through binding to mRNA targets; however, it is a molecule consisting of multiple copies of mature miRNAs (Daugaard and Hansen, 2017).

All biogenesis pathways result in the formation of mature miRNA molecules and consequent assembly of the miRISC complex. This ribonucleoprotein complex serves as a catalytic engine for the posttranscriptional regulation of gene expression, during which miRNA binds to particular mRNA via its seed region at the 5′-end of the molecule. Proteins of the miRISC complex, such as Ago, Snd1, and MTDH, are responsible for translation inhibition, mRNA target degradation, and promotion of the protection of miRNA from the action of intracellular nucleases, determining the duration of life and functional period of mature miRNA (Kim et al., 2009). The key interaction for assembling and function of miRISC complex is appeared to be co-operation of scaffold protein TRNC6B and protein with ribonuclease activity Ago2. Interactions between tryptophan residues in Ago-binding domain (ABD) of TRNC6B and tryptophan-binding pockets of Ago2 were recently discovered to promote the phase transition (Sheu-Gruttadauria and MacRae, 2018). This process represents formation of viscoelastic TRNC6B - Ago2 droplets heterogeneous on the atomic level since Ago2 interacts not only with rigorously defined tryptophan residues but also with random indole groups of tryptophans in ABD of TRNC6B. Apart from aforementioned proteins different miRNAs and their direct mRNA targets are included in the composition of droplets during the phase transition. It was evaluated that only one couple of miRNA–mRNA is introduced in the droplet suggesting the occurrence of selection process for particular mRNA molecules (Sheu-Gruttadauria and MacRae, 2018). *In vitro* experiments demonstrated that TRNC6B - Ago2 droplets are formed to degrade mRNAs (Sheu-Gruttadauria and MacRae, 2018). It becomes possible since Ago2 protein retains its catalytic activity and is able to promote target mRNA slicing. In addition, the active subunits of de-adenylation CCR4-NOT complex were also found in the droplets aimed at mRNA de-adenylation after its slicing (Sheu-Gruttadauria and MacRae, 2018). Thus, novel data give evidence that mRNA degradation following binding with miRNA occurs in separate heterogeneous viscoelastic structures that concentrate particular mRNA targets and protein components for its degradation. These results turn around the present perceptions about miRISC complex opening the new horizons for miRNA-based investigations. In particular, it raises a question about existence of special structural organization of miRNAs and their mRNA targets in cell cytoplasm necessary for the regulation of its function and decay.

## Intracellular miRna Turnover

While the biogenesis and function of miRNAs are described in detail, the mechanism of cellular miRNA degradation remained unclear until relatively recently. Since 2010, increasing amounts of data have become available regarding miRNA degradation as a result of miRNA binding to non-coding RNAs (Ameres et al., 2010). In particular, it has been shown that the level of miRNA-27 falls significantly following binding to viral *H. samiris* U-rich non-coding RNAs or the m169 transcript that was defined as natural inhibitor of miRNA-27, which enter the cell as a result of infection with *Herpesvirus Samiri* or *Cytomegalovirus*, respectively (Cazalla et al., 2010; Marcinowski et al., 2012). It has been stressed that such a phenomenon is accompanied by the elongation of miRNA with several U or A nucleotides, followed by 3′-end trimming of the miRNA molecule. This process is called target RNA-directed miRNA degradation (TDMD), and in confirmation of such a mechanism, several enzymes, namely terminal uridine-transferases (TUT) and 3′-exonucleases (for example, DIS3L2), have been found to take part in TDMD and carry out the transfer of uridines to the 3′-end of miRNA and the consequent miRNA 3′-trimming, respectively (Haas et al., 2016; Figure 1). The most important parameter determining the efficiency of TDMD is the degree of complementarity between the miRNA and its RNA target. It has been identified that the tight binding between the 3′-end of miRNA and the 5′-end of its target must occur for the initiation of TDMD. A 2-nt mismatch may cause greater than a three-fold decrease in the efficiency of TDMD, and a 4-nt mismatch may completely abolish miRNA degradation. Moreover, if there is a central bulge in the miRNA/RNA target complex, it should comprise no more than 5 nt, otherwise TDMD will not take place (De la Mata et al., 2015). It is likely that the different levels of complementarity between mammalian miRNAs and their RNA targets promoted separation of the pathways of TDMD and miRNA-mediated posttranscriptional regulation of gene expression. It should be stressed that conventional binding of target mRNA to the miRNA seed region does not initiate miRNA degradation due to the low degree of complementarity (Ameres et al., 2010).

Another factor that may determine the balance between TDMD and target RNA decay is miRNA abundance. According to a study in neuronal cells, low-abundance miRNAs, for example miRNA-132, bind to RNA targets with high complementarity and initiate TDMD, whereas highly abundant miRNAs, such as miRNA-138, miRA-128, and miRNA-124, mediate target RNA decay. Confirmation of such a phenomenon showed that the high intensity of TDMD observed for miR-132 fell dramatically after the level of miRNA-132 was elevated following transfection with synthetic mimics (De la Mata et al., 2015).

Finally, its non-cumulative nature is the last important feature of TDMD. An increase in the number of binding sites for miRNA within target mRNA significantly improves its inhibitory effect but does not stimulate TDMD. To illustrate, it has been shown that the addition of miRNA binding sites to the m169 transcript structure has no impact on TDMD efficiency (Haas et al., 2016); however, a two-fold decrease in the miRNA-132 degradation rate was observed following an increase in the number of binding sites in the RNA target from one to four (De la Mata et al., 2015).

Thus, TDMD represents a non-cumulative degradation of miRNA accomplished by two enzymes and resulting from miRNA binding to full-length complementary targets. It should be noted that terminal uridine transferases and 3′-exonucleases that carry out TDMD interact with Ago family proteins (Haas et al., 2016), suggesting that there is co-regulation of the miRNA biogenesis, functioning, and degradation processes.

## The Role of miRnas in Oncopathologies. Oligonucleotide-Based Approaches to the Modulation of miRna Function

Recently, a vast amount of studies have been published stating the direct involvement of miRNAs in malignant growth. Aberration of miRNA expression is not uncommon during the initiation and progression of various diseases, including cancer (Rupaimoole et al., 2016; Singh and Sen, 2017). Investigators have discriminated between oncogenic miRNAs that promote tumor development and tumor suppressor miRNAs that impede tumorigenesis (Nucera et al., 2016; O’Bryan et al., 2017). Oncotransformation and metastasis during each step can be accompanied by the downregulation of tumor suppressor miRNAs and the hyperexpression of oncogenic miRNAs. During the initiation stage, associated mainly with uncontrolled cell proliferation, clusters of oncogenic miRNAs, in particular miRNA-17-92 and miRNA-106b, appear to be the main regulators (Tan et al., 2014; Li H. et al., 2017). Cell immortalization, which involves intense cell division and evasion of apoptosis, may be partly connected to the action of such oncogenic miRNAs: miRNA-125b that plays a role in the inhibition of apoptosis; miRNA-221/222 that enhance the proliferative potential of cells; miRNA-130b that provides resistance to chemotherapy; and miRNA-21 that exhibits a complex pathological influence on various cellular functions (Masoudi et al., 2018; Zhang et al., 2018). Substantial vascularization, which furnishes the forming neoplasia with ceaseless nutrition, is the consequence of the angiogenic action of multiple miRNAs, including miRNA-9, miRNA-27b, miRNA-130a, miRNA-210, miRNA-191, and miRNA-378 (Markopoulos et al., 2017). However, the process of tumor cell dissemination (metastasis), is prompted by the activity of miRNA-155, miRNA-9, miRNA-10b, and miRNA-21 (Devulapally et al., 2015; Mignacca et al., 2016), during which, miRNA-181b, miRNA-193a, and miRNA-29a are also engaged in modulating the adhesive properties of cells (Mohamad et al., 2016; Liu et al., 2017). Currently, manipulation of miRNAs associated with tumorigenesis is of considerable scientific and practical interest.

**FIGURE 2:**
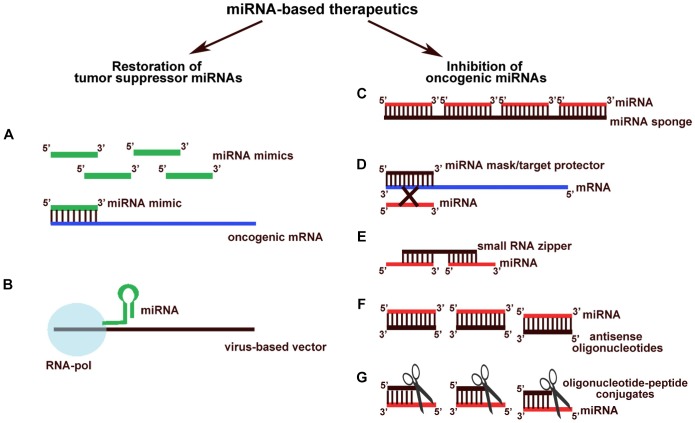
Different oligonucleotide-based miRNA-targeted therapeutics. Constructions aimed at restoration of levels of tumor suppressor miRNAs: **(A)** synthetic miRNA mimics; **(B)** endogenously expressed tumor suppressor miRNAs from virus-based vectors. Therapeutics aimed at inhibition of oncogenic miRNAs: **(C)** miRNA sponges; **(D)** miRNA masks (target protectors); **(E)** small RNA zippers; **(F)** antisense oligonucleotides; **(G)** oligonucleotide-peptide conjugates (miRNases).

Among the ways by which to modulate the level of a particular miRNA, restoration of tumor suppressor miRNA by straightforward transfection of synthetic miRNA mimics (Zhang Y. et al., 2016; Tian et al., 2017) or transformation of cells with vectors expressing deficient miRNAs has been investigated in most depth (Michelfelder and Trepel, 2009), and a number of positive results have already been obtained in this area (Figure 2A,B). Transfection of acute promyelocytic leukemia cells with synthetic miRNA-218 reduces viability, inhibits intracellular DNA synthesis, promotes cell cycle arrest in the G_0_/G_1_ phase, and triggers apoptosis, leading to a two-fold increase in the number of apoptotic cells (Wang Y. et al., 2017). Delivery of miRNA-1193 and miRNA-455 decreases the invasive potential of breast cancer and non-small cell lung cancer up to three times as compared with the control (Li et al., 2016; Li X. et al., 2017). Treatment of nasopharyngeal carcinoma, colorectal cancer, and non-small lung cancer with synthetic miRNA-497, miRNA-495, and miRNA-142, respectively, leads to a 2.5-fold suppression of migration and proliferation of tumor cells and a two-fold tumor growth retardation *in vivo* (Wang et al., 2015; Wang Z. et al., 2017; Yan et al., 2017). Expression of miRNA-26 in an adenoviral vector inhibits tumorigenesis, induces apoptosis, and suppresses cancer cell growth in a model of Myc-dependent liver cancer (Kota et al., 2009). Expression of miRNA-15a in a lentiviral vector considerably reduces colony formation and proliferation of endometrial cancer cells (Wang Z.M. et al., 2017).

Restoration of tumor suppressor miRNA levels for the treatment of oncopathologies represents a relatively promising strategy. À number of therapeutics based on miRNA mimics are currently undergoing clinical trials. TargomiRs, which represent minicells containing an miRNA-16 mimic for malignant pleural mesothelioma treatment, successfully passed Phase I clinical trials by EnGeneIC Ltd. (Van Zandwijk et al., 2017). In 2013, clinical trials for MRX34 – a synthetic miRNA-34a mimic – were initiated; however, regrettably, in 2017, Mirna Therapeutics Inc., ascertained that this agent promotes adverse events (Beg et al., 2017). The recruitment of patients for Phase II clinical trials of the drug, MRG-201, which represents the mimic of miRNA-29 initiated by miRagen Therapeutics Inc., is currently being executed (ClinicalTrials.gov Identifier: NCT03601052). Despite being aimed at the treatment of keloids, such a therapeutic agent may be tested as an antitumor vehicle, since miRNA-29 plays a relevant tumor suppressive role in various cancers, including myeloid leukemia, esophageal squamous cell carcinoma, and gastric cancer (Qi et al., 2017).

Based on the application of synthetic constructions, a number of technologies have been developed to inhibit the hyperfunctions of oncogenic miRNAs. Such compounds may be aimed at both direct miRNA sequestration and interruption of miRNA regulatory activity through the interaction with mRNA targets, but not at the miRNAs themselves. For instance, miRNA sponges induce a significant decrease in the level of functionally active oncogenic miRNAs operating as additive sites for miRNA binding (Tay et al., 2014; Figure 2C). Many sponge constructs have been developed to inhibit various miRNAs, including miRNA-10b, miRNA-21, miRNA-19, miRNA-155, miRNA-23b, miRNA-221/222, miRNA-9, and miRNA-140 (Haraguchi et al., 2009; Ma et al., 2010; Chen et al., 2013; Liu et al., 2014; Mignacca et al., 2016). The *in vitro* effects of their application are: a 40% decrease in the invasive and migratory potentials of tumor cells (Liang et al., 2016); up to a 50% inhibition of cell proliferation (Mignacca et al., 2016); and a two-fold increase in the sensitivity to chemotherapeutics, in particular doxorubicin (Gao et al., 2015). *In vivo* administration of miRNA sponges results in a reduction in angiogenesis and a two-fold reduction in the number of metastases formed in the case of glioma and colorectal cancer (Ma et al., 2010; Liu et al., 2014).

Other antisense oligonucleotide-based agents for miRNA-mediated therapy are miRNA masking oligonucleotides or target protectors, which impede the binding of oncogenic miRNAs to their targets via an interaction with the 3′-untranslated region of mRNAs followed by the reactivation of normal activity of genes that were previously repressed (Choi et al., 2007; Figure 2D). In 2012, miRNA masking oligonucleotides were used for the first time to investigate miRNA function in tumorigenesis. These experiments resulted in the evaluation of the stimulatory impact of TP63 protein expression on breast cancer cell proliferation, which proved to be directly regulated by miRNA-196a2^∗^ (Kim et al., 2013). Since then, the functional relationships between dozens of miRNAs and their mRNA targets have been ascertained by means of miRNA masking oligonucleotides, including miRNA-203 and the *LASP-1* (LIM and SH3 Protein 1) gene, miRNA-17 and miRNA-20à and the *NOR-1* (Neuron-derived Orphan Receptor-1) gene, miRNA-29-b-1 and the *SPIN1* (Spindlin 1) gene, and miRNA-27à and the *CALR* (calreticulin) gene. These interactions appear to be crucial for tumorigenesis events such as an increase in the invasive, proliferative, angiogenic, and migratory potentials, and evasion of immunogenic apoptosis (Colangelo et al., 2016; Drago-Ferrante et al., 2017). Evidence highlights the promising application of miRNA masking oligonucleotides as inhibitors of oncogenic miRNA function in tumor cells. For instance, effective proliferation suppression and apoptosis induction have been described for miRNA masks that prevent the interaction of miRNA-522 with *DENND2D* mRNA; the negotiation of glioblastoma cell resistance to temozolomide has been evaluated using target protectors that impede the interplay between miRNA-9 and *PTCH1* mRNA; and the inhibition of angiogenesis has been achieved by blocking the binding of the miRNA-30 family to *DLL4* (Delta-like 4) mRNA (Bridge et al., 2012; Munoz et al., 2015; Zhang T. et al., 2016). However, despite this progress and promising *in vitro* results, there still exists no data regarding the application of miRNA masking oligonucleotides *in vivo*.

In 2017, a novel type of oligonucleotide construction, named small RNA zippers, was designed, which can block miRNA function by forming a duplex with multiple copies of miRNAs, linking them together in an end-to-end manner (Meng et al., 2017; Figure 2E). *In vitro* application of zippers targeted to oncogenic miRNA-17 and miRNA-221 in a breast cancer cell line has been shown to decrease the level of targeted miRNAs by up to 90%, followed by the inhibition of cancer cell migration and a 1.5-fold increase in the sensitivity to doxorubicin (Meng et al., 2017). This is the only article that states the application of small RNA zippers; however, these first data show a high therapeutic potential of such constructions, the biological effect of which is comparable with the efficiency of current antisense oligonucleotides.

Another technique becoming increasingly popular nowadays is application of clustered regularly interspaced short palindromic repeats (CRISPR)-associated nuclease 9 (Cas9) – CRISPR/Cas9 systems. They consist of Cas9 endonucleases cloned from *Streptococcus pyogene* and single guide RNA (sg RNA). The latter, in turn, represents two sequences: (1) CRISPR RNA (crRNA) that is complementary to target DNA sites and responsible for its recognition and binding; and (2) *trans*-activating CRISPR RNA (trancrRNA) that is partially complementary to crRNA and is essential for maintenance of Cas9 nuclease activity. The CRISPR/Cas9 systems distinguish target DNA sequences that are located in direct proximity to protospacer adjacent motif (PAM) and induce the double-stranded breaks. Hereafter, the repair system patches the breaks in non-homologous end joining manner with variable sizes of insertions or deletions followed by inhibition of target molecule expression and activity. To suppress functions of oncogenic miRNA, investigators usually apply CRISPR/Cas9 systems that introduce mutations to the Drosha/Dicer processing site of miRNA precursors (pri- or pre-miRNAs) that leads to the cancelation of further biogenesis and decrease in the level of mature miRNA in cells, in average, by 55–96% (Yang et al., 2018). The consequences of inhibition of oncogenic miRNAs such as miRNA-17, miRNA-21, miRNA-141 and miRNA-3188 using specific CRISPR/Cas9 systems are 1.5-2-fold inhibition of proliferation and invasion, up to 5-fold decrease in migration as well as two-fold effective induction of cancer cells apoptosis as compared to control (Aquino-Jarquin, 2017; Huo et al., 2017). In addition, the CRISPR/Cas9 was found to inhibit epithelial-mesenchymal transition and significantly increase the sensitivity of tumor cells to chemotherapeutics, in particular, cisplatin and paclitaxel (Aquino-Jarquin, 2017; Huo et al., 2017). CRISPR/Cas9 systems is out of question the perspective approach to downregulate the oncogenic miRNAs and though its effectiveness may vary for different miRNA targets, this feature is balanced out by high stability and duration of its effect that may last 30 days for both *in vitro* and *in vivo* models (Chang et al., 2016). Moreover, it should be noted that increased specificity of CRISPR/Cas9 turns it to the highly precise instrument for inhibition of particular oncogenic miRNAs from one miRNA cluster.

One of the promising approaches for decreasing the hyperexpression of miRNAs is the application of synthetic antisense oligonucleotides (or anti-miRNA ONs; Figure 2F). Antisense oligonucleotides are single-stranded DNAs, 15-20 nt in length, which have historically been used to inhibit mRNA targets and are currently applied to modulate the activity of miRNAs. Antisense oligonucleotides function as competitive inhibitors of miRNAs, which complementarily interact with mature molecules and cause steric hinderance of function or complete degradation mediated by RNase H (Boutla et al., 2003). A number of modifications of antisense oligonucleotides, including structural changes to the sugar backbone such as 2′O-Methyl (2′OMe), 2′-Fluoro (2′F), 2′O-Metoxyethyl (2′MOE), Locked Nucleic Acids (LNA), and Peptide Nucleic Acids (PNA), as well as chemical modifications to the phosphodiester bonds, in particular phosphorothioate (PS) and N-mesyl- (μ-) and methoxyethyl-phosphoramidate have been developed, allowing a significant increase in the therapeutic potential of antisense oligonucleotides via the enhancement of nuclease resistance and binding affinity, and facilitation of penetration across the cell membrane (Lennox and Behlke, 2010; Watts, 2013; Miroshnichenko et al., 2019; Supplementary Figure S1). Under the action of different chemically modified antisense oligonucleotides, it is possible to attain a considerable reduction in oncogenic miRNA activity, and consequently to inhibit various events of tumorigenesis. For instance, the inhibition of oncogenic miRNAs, such as miRNA-23a and miRNA-106b∼25 cluster, has been found to promote a decrease in the proliferative potential of cancer cells by up to 75% (Zhang R. et al., 2016; Quan et al., 2017). In addition, application of antisense technology may cause an approximate two-fold decrease in cellular motility and a two-fold increase in the percentage of apoptotic cells following transfection with anti-miRNA ONs targeted, in particular, to miRNA-21, miRNA-221, miRNA-17, miRNA-18a, and miRNA-191 (Haghpanah et al., 2016; Liang et al., 2017; Quan et al., 2017). Experiments in breast cancer cells, glioblastoma cells, and medulloblastoma cells have shown that the anti-metastatic and anti-apoptotic effects of oligonucleotides targeted to miRNA-191, miRNA-10b, miRNA-221, or miRNA-222 are two-fold higher (Quintavalle et al., 2011; Pal and Greene, 2015; Sharma et al., 2017). *In vivo* administration of antisense oligonucleotides has been found to reduce tumor growth (Mercatelli et al., 2008; Li et al., 2009). For instance, ONs targeted to miRNA-10b cause greater than a two-fold retardation of tumor growth and a considerable increase in the survival of mice with intracranial glioblastoma (Tepluyk et al., 2016). Administration of anti-miRNA-155 ONs to mice suffering from lymphoma led to a five-fold reduction in tumor weight as compared with the control (Babar et al., 2012). Moreover, antisense oligonucleotides may also have an inhibitory effect on metastasis *in vivo*. Administration of anti-miRNA-182 ONs was shown to promote a two-fold decrease in the number of liver metastases at the latest stage of melanoma (Huynh et al., 2011), while inhibition of miRNA-10b led to complete elimination of metastases in the lymph nodes of xenograft mice with breast cancer (Yoo et al., 2017). Furthermore, antisense miRNA-based oligonucleotides may also suppress angiogenesis and stimulate tumor infiltration by macrophages *in vivo* (Kong et al., 2014). In addition, antisense oligonucleotides may serve as effective therapeutic agents to treat pathologies which require overcoming the blood-brain barrier including glioblastoma and neuro-degenerative diseases. Initially, antisense oligonucleotides were not supposed to be delivered to brain systematically, but to date the significant progress in the area of delivery of such agents beyond the blood-brain barrier has been achieved. Two main types of delivery provided passing through the blood-brain barrier are well investigated today, in particular, direct and peripheral delivery. Direct methods include intratumoural or intraventicular administration of anti-miRNA ON using osmotic pomp that is effective in terms of glioblastoma treatment (Kim et al., 2016). Peripheral delivery of antisense oligonucleotides implies (1) subsequent administration of ONs with agents that contribute in re-arrangement of blood-brain barrier such as angubudin-1 leading to transient dissociation of proteins from tight junctions of blood brain barrier followed by size-selective leakage of therapeutic ON; or (2) conjugation of ONs with different groups, such as ligands to the receptors on the surface of blood-brain barrier or arginine-rich cell penetrating peptides to cross the barrier by receptor-mediated endocytosis, inverted micelle or pore formation as well as direct membrane translocation depending on the type of conjugated group (Evers et al., 2015; Zeniya et al., 2018). All these approaches showed high specificity and effectiveness *in vivo* without any adverse effects. Successful application of antisense oligonucleotides *in vivo* gave an impetus to initiate clinical trials. MRG-106 (miRagen Therapeutics, Inc.), an anti-miRNA-155 drug for the treatment of a wide spectrum of leukemias and lymphomas, and RG012 (Regulus Therapeutics Inc.), an oligonucleotide targeted to miRNA-21 for curing Alport syndrome, have already entered clinical trials. Thus, antisense technology represents a highly effective and rapidly progressing area of research related to miRNA.

Finally, one of the recent directions in miRNA-based therapy is the application of artificial ribonucleases (aRNases) that are conjugates of miRNA-targeted oligonucleotides and catalytic constructs (Gaglione et al., 2011; Danneberg et al., 2015; Patutina et al., 2017, 2018; Figure 2G). One of the variants of effective constructions is miRNA-targeted metal-dependent synthetic RNases that represent conjugates contained (1) peptide nucleic acids (PNA) for highly effective target RNA binding and (2) diethylenetriamine (DETA) or a three-amino acids peptide HGG with Cu^2+^ ion, as a co-factor, aimed at the oxidative or acid-base cleavage of hsa-miR-1323, which is involved in the development of neuroblastoma (Gaglione et al., 2011). Another aRNase targeted to oncogenic miRNA, in particular miR-20a from oncogenic cluster miRNA-17∼92, comprises metal-independent artificial RNase consisting of a PNA oligonucleotide with tris(2-aminebenzimidazole) which degrades miRNA substrate, presumably, in RNase A-like manner in the bulge forming upon the hybridization (Danneberg et al., 2015). To date, the most successful miRNA-based conjugates, miRNAses, represent conjugates of oligonucleotides and the catalytic peptide [(LRLR)G]_2_ that cleave clinically relevant miRNA targets, miRNA-21 and miRNA-17 by *trans*-esterification reaction (Patutina et al., 2017, 2018). These miRNases have shown a significant reduction in target miRNA levels in tumor cells, followed by the restoration of key tumor-suppressor proteins and considerable inhibition of cell proliferation without any off-target effects.

Currently established miRNA-based therapeutics represent a spectrum of oligonucleotide constructions with different mechanisms of oncogenic miRNA activity modulation (Figure 2). In particular, miRNA sponges, small RNA zippers, and some types of antisense oligonucleotides that do not exhibit RNase H-activating ability (2′OMe, LNA, PNA etc.) effectively suppress the activity of oncogenic miRNAs by forming a steric duplex. MiRNA masks act as competitive inhibitors of miRNAs by allowing restriction of the influence of one separated miRNA on a particular target via complementary binding to corresponding mRNAs. CRISPR/Cas9 systems downregulate miRNAs by inclusion of mutations to the miRNA precursors sequences and prevention of miRNA biogenesis. Antisense oligonucleotides that are recognized by RNase H, in addition to aRNases, irreversibly degrade target miRNAs and effectively inhibit their function in cells. These approaches have demonstrated the relatively high *in vitro* and *in vivo* efficiency and have laid the foundation for further investigation of these miRNA-based strategies in combined applications.

**Table 1:** Mixes of miRNA-based therapeutics that exhibit a synergistic effect on tumorigenesis *in vitro* and *in vivo.*

Therapy	Concentration of each compound in the mix	*In vitro/ex vivo/ in vivo*	Cellular function undergoing synergistic influence	Sum of effects of separate agent treatment^∗^, fold^∗∗^	Effect of combined therapy, fold^∗∗^	References
Anti-miRNA-183, anti-miRNA-182 and anti-miRNA-96 ONs	0.4 μM	*In vitro*	Proliferation and colony formation inhibition	2.6-fold – proliferation inhibition, 7-fold – colony formation inhibition	5-fold – proliferation inhibition, complete inhibition of colony formation	Zhang et al., 2015a
Anti-miRNA-221 and anti-miRNA-222 R8 conjugates	1 μM	*In vitro*	Apoptosis induction	2.7-fold	6-fold	Brognara et al., 2015
Anti-miRNA-21 and anti-miRNA-10b	100 nM	*In vitro*	Invasion inhibition	3.8-fold	7-fold	Dong et al., 2012
miRNA-34a and miRNA-let-7b mimics	25 or 50 nM	*In vitro*	Invasion inhibition	1.72-fold	5.5-fold	Kasinski et al., 2014
miRNA-126 and miRNA-34a mimics	1 pfu/cell (virus vector)	*In vitro*	Apoptosis induction	4.75-fold	6.3-fold	Feng et al., 2017
miRNA-497 and miRNA-34a mimics	50 nM	*Ex vivo*	Tumor growth inhibition	4.75-fold	9-fold	Han et al., 2015
miRNA-634 and temozolomide	25–400 μM temozolomide, miRNA mimic – N/A	*In vitro*	Colony formation inhibition	3.6-fold	5.6-fold	Tan et al., 2018
miRNA-205 mimic and gemcitabine	40 mg/kg gemcitabine, miRNA mimic – N/A	*Ex vivo*	Tumor growth inhibition	4.3-fold	6.1-fold	Chaudhary et al., 2017
Anti-miRNA-21 and gemcitabine	2 mg/kg gemcitabine, 80 μg/mice ON	*In vivo*	Tumor growth and metastases inhibition	4-fold tumor growth inhibition, 3-fold decrease in metastases	5.5-fold tumor growth inhibition, complete elimination of metastases	Li Y. et al., 2017
miRNA let-7b mimic and paclitaxel	50 nM mimic, 25 nM paclitaxel – *in vitro*; 1 mg/kg mimic, 5 mg/kg paclitaxel	*In vitro/in vivo*	Apoptosis induction, tumor growth inhibition	7.8-fold apoptosis induction, 3-fold tumor growth inhibition	13.9-fold apoptosis induction, 5.7-tumor growth inhibition	Dai et al., 2016
miRNA-34a mimic and celecoxib	100 μM celecoxib, miRNA mimic – N/A	*In vitro*	Migration inhibition	3.6-fold	5.1-fold	Chen et al., 2017
miRNA-218 mimic and temozolomide	10 mg/kg temozolomide, miRNA mimic – N/A	*In vivo*	Tumor growth inhibition	10.5-fold	42-fold	Fan et al., 2015

*^∗^Sum of effects of separate agent treatment is the sum of effectiveness observed during monotherapy with each agent. ^∗∗^Fold change was calculated as the ratio of effectiveness of therapeutic agent and the control compound. N/A – concentration of miRNA-based therapeutics applied in the study is not mentioned.*

## Effects of the Simultaneous Application of Several miRna-Based Therapeutics on Tumorigenesis *In Vitro* and *In Vivo*

In the present review, three strategies for the combined application of miRNA-based therapeutics are considered: (1) simultaneous application of more than one antisense oligonucleotide targeted to oncogenic miRNAs; (2) transfection of tumor suppressor miRNA mimic combinations; (3) treatment with antisense oligonucleotides or mimics in conjunction with chemotherapeutics. Supplementary Table S1 summarizes the results of reported preclinical studies on the combined application of miRNA-based therapeutics *in vitro* and *in vivo*. This table contains a detailed description of all the effects of mono- and combination therapy for each individual study described below, with a view to keeping track of the efficiency of combination therapy. The most impressive results showing a synergistic effect of combination therapy are listed in Table 1.

In *in vitro* experiments, the final concentration of the applied ON mix was mainly 50 or 100 nM (Lee et al., 2015; Zhang R. et al., 2016; Li Y. et al., 2017); however, in some cases, the concentration of the mix was significantly lower (10 or 30 nM; Matsubara et al., 2007; Lu et al., 2009) or, vice versa, reached 1.2-2 μM (Brognara et al., 2015; Zhang et al., 2015a), since such combinations were applied in the absence of any delivery vehicle. It should be stressed that the concentration of each oligonucleotide in the mix was less than the final concentration in proportion to the number of compounds used.

Cancer cells were transfected with pre-miRNAs or miRNA mimics at a concentration of 10-50 nM each (Cheng et al., 2017; Zeng et al., 2018; Jiang et al., 2019), and only in one case of concurrent application of an miRNA mimic and chemotherapy was the concentration of the miRNA mimic 200 nM (Huang et al., 2014).

In combination with miRNA-based agents, chemotherapeutics were applied at different concentrations depending on the cancer cell type: temozolomide was used to treat glioma and glioblastoma at 25-400 μM (Tan et al., 2018; Zeng et al., 2018); gemcitabine was applied to various types of pancreatic cancer cell lines at concentrations of 500 nM to 20 μM (Chaudhary et al., 2017; Tu et al., 2019); sunitinib was used to treat glioblastoma cells at 15 or 20 μM (Costa et al., 2013; Liu et al., 2015), as well as at 1 μM in the case of kidney cancer cell treatment (Khella et al., 2015); and cetuximab was added to colorectal cancer and hepatocellular carcinoma cell lines at 10-13 μM (Huang et al., 2014; Zhou et al., 2015).

*In vivo* experiments were conducted using miRNA-based therapeutics at doses from 1 to 4 mg/kg (Mittal et al., 2014; Dai et al., 2016; Li Y. et al., 2017) and chemotherapies were administrated at the following doses: gemcitabine, 40 mg/kg (Chaudhary et al., 2017; Mondal et al., 2017); temozolomide, 10-20 mg/kg (Fan et al., 2015; Zeng et al., 2018); paclitaxel, up to 5 mg/kg (Dai et al., 2016; Tu et al., 2019); and sunitinib, 30 mg/kg (Costa et al., 2015).

## Combination of Anti-miRna Oligonucleotides for Overall Inhibition of Oncopathological States

The relatively high biological activity of a particular anti-miRNA oligonucleotide *in vitro* and *in vivo* suggested that combined administration of several miRNA-targeted agents may reinforce the antitumor effect. Two strategies have proven to be effective. The first is related to the application of several oligonucleotides targeted to multifunctional oncomiRs, such as miRNA-155, miRNA-21, and miRNA-10b, which modulate multiple events of tumorigenesis (Pfeffer et al., 2015; Wang, 2017). The second strategy includes co-inhibition of miRNAs that belong to one oncogenic miRNA cluster, which usually combines mono- and polyfunctional regulators, as in the case of the miRNA-183-96-182 cluster, where miRNA-96 mostly stimulates cell proliferation, whereas polyfunctional miRNA-182 and miRNA-183 intensify the invasive and migratory potentials of cancer cells and promote chemotherapy resistance (Ren et al., 2014; Yan et al., 2014; Zhang et al., 2015c; Ma et al., 2016).

**FIGURE 3:**
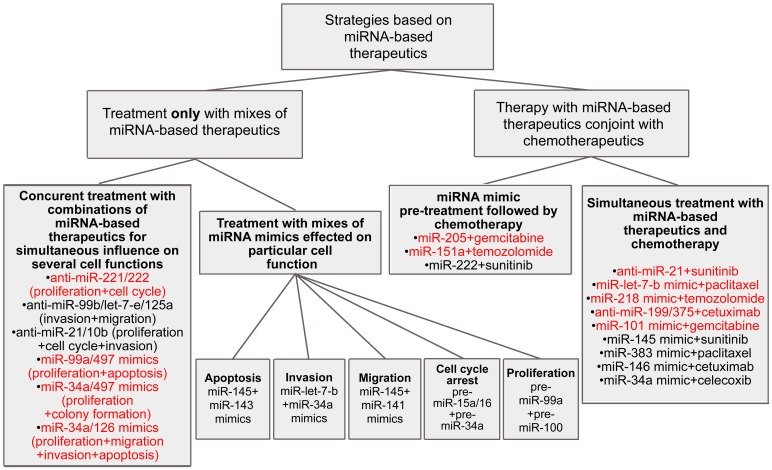
Different combinative strategies to inhibit tumorigenesis. Mixes exhibited high efficiency *in vivo* are colored by red.

Treatment of cancer cells with various oligonucleotide mixes has resulted in considerable enhancement of therapy efficiency in comparison with the effects of a single anti-miRNA ON. Simultaneous silencing of miRNA-17 and miRNA-20à in the miRNA-17∼92 cluster or three miRNAs in the miRNA-183/182/96 cluster leads to a 3- or 4-fold greater inhibition of human colon and lung cancer cell viability (Matsubara et al., 2007; Zhang et al., 2015a; Table 1 and Supplementary Table S1). A combination of miRNA-130a and miRNA-495 antisense inhibitors promotes a two-fold greater inhibition of angiogenesis in gastric cancer (Lee et al., 2015; Supplementary Table S1). Application of cocktails containing oligonucleotides targeted to miRNAs from the miRNA-106b∼25 cluster, including miRNA-106, miRNA-93, and miR-25, and miRNAs from the miRNA-221/222 cluster, leads to a 1.5-fold to 4-fold greater rate of apoptosis induction than with each oligonucleotide separately (Brognara et al., 2015; Zhang R. et al., 2016; Table 1 and Supplementary Table S1). Moreover, various mixes of anti-miRNA oligonucleotides, in particular anti-miRNA-221 and anti-miRNA-222, and anti-miRNA-10b and anti-miRNA-21, as well as anti-miRNA ONs to all miRNAs from the miRNA-106b∼25 cluster, are two-fold stronger in terms of decreasing the proliferative potential of glioma and glioblastoma cells (Zhang et al., 2009, Dong et al., 2012; Zhang R. et al., 2016; Table 1 and Supplementary Table S1). A reduction in the invasive and migratory potentials as great as 3.5-fold may be reached using mixes of anti-miRNA-99b, anti-miRNA-let-7-e, and anti-miRNA-125a oligonucleotides or anti-miRNA-21 and anti-miRNA-10b oligonucleotides in comparison with monotherapy (Dong et al., 2012; Ma et al., 2017; Table 1 and Supplementary Table S1). It should be noted that in most cases, oligonucleotide cocktails have a simultaneous influence on several different events of tumorigenesis (Li L. et al., 2014; Zhang R. et al., 2016; Ma et al., 2017; Figure 3).

Successful application of antisense inhibitor mixes promoted the development of oligonucleotide constructs able to provide combined delivery of anti-miRNA agents to cells. Two very similar structures were independently established, namely multitarget antisense oligonucleotides (MTg-AMO) and multipotent miRNA sponges, which represent long oligonucleotides containing several sites for binding to the miRNA of interest. The substantial difference between these inhibitors is the method of intracellular delivery, with MTg-AMOs being co-transfected with delivery agents including lipofectamine, and miRNA sponges being expressed using viral vectors. Application of an miRNA-21/miRNA-155/miRNA-17 MTg-AMO and an miRNA sponge complementary to miRNA-21, miRNA-155, miRNA-221, and miRNA-222 were found to considerably decrease cancer cell viability and proliferation, with the effect of multitarget inhibitors being up to 60% more effective than that of separate inhibitors (Lu et al., 2009; Jung et al., 2015; Supplementary Table S1). Moreover, using a miRNA sponge targeted to miRNA-17, miRNA-18a, miRNA-19, and miRNA-92, it is possible to attain up to a 6-fold greater inhibition of lymphoma cell growth as compared with the effect of miRNA sponges targeted to individual miRNAs (Kluiver et al., 2012; Supplementary Table S1). In addition, a significant increase in the percentage of apoptotic cells may be reached by the application of multipotent sponges targeted to the miRNA-183-96-182 cluster or containing simultaneous binding sites for miRNA-17 and miRNA-20a (Li P. et al., 2014; Niu et al., 2014).

*In vitro* results of the application of oligonucleotide cocktails targeted to oncogenic miRNAs are relatively promising. The most outstanding of these proves that the application of anti-miRNA oligonucleotide mixes results in a 4- to 7-fold decrease in the invasive potential of tumor cells (Dong et al., 2012; Zhang R. et al., 2016; Supplementary Table S1), up to a 5-fold induction of apoptosis of cancer cells (Brognara et al., 2015; Supplementary Table S1), and up to a 9-fold suppression of cancer cell growth (Kluiver et al., 2012; Supplementary Table S1); whereas the average pro-apoptotic and anti-proliferative effects of single anti-miRNA oligonucleotide therapy do not exceed 20-25%. Nevertheless, there is only one publication that demonstrates the *in vivo* application of an antisense oligonucleotide mix that consisted of anti-miRNA-221 and anti-miRNA-222 ONs, and provided only a 1.5-fold greater inhibition of tumor growth as compared with each ON used alone (Zhang et al., 2009; Supplementary Table S1).

## Application of Synthetic miRna Mimic Mixes for an Enhanced Effect on a Particular Cellular Function

Another approach for the creation of combinations of miRNA-based therapeutics is the concurrent application of synthetic miRNA mimics. The most often used are mimics of miRNA-34a, miRNA-99a, and members of the miRNA-145 family. The employment of such synthetic miRNA mimics arose from the key regulatory roles of these tumor suppressor miRNAs in cell cycle surveillance, focal adhesion, and cytokine-cytokine receptor interactions in cancer cells of various histogenesis via adjustment of fundamental signaling pathways including p53, Notch, TGF-β, IGF-1R, and mTOR (Li and Zheng, 2017; Huang et al., 2018; Ren et al., 2018). Therefore, restoration of normal expression levels of the proteins involved in these pathways and regulated by these miRNAs may account for normalization of cellular processes and inhibition of the development of pathological conditions.

In contrast to the application of antisense oligonucleotide mixes that exert a complex influence on several events of tumorigenesis simultaneously, miRNA mimic combinations more frequently exhibit a considerable effect on one particular cellular function (Figure 3). For instance, simultaneous treatment of non-small lung cancer cells with pre-miRNA-15a/16 and pre-miRNA-34a promotes an increase in the number of cells undergoing arrest in the G1-G0 phase of the cell cycle in a synergistic manner; approximately 55% of cells are under arrest, which is almost three-fold greater than the results seen with monotherapy (Bandi and Vassella, 2011; Supplementary Table S1). However, a synergistic pro-apoptotic effect of such a mix is not observed. Simultaneous transfection of non-small cell lung cancer cells with miRNA-34a and miRNA let-7b mimics promotes a significant decrease in invasive potential: the number of invading cells is 5- to 8-fold less, as compared with the application of let-7b or miRNA-34a mimics alone, respectively (Kasinski et al., 2014; Table 1 and Supplementary Table S1). Application of a cocktail consisting of pre-miRNA-145 and pre-miRNA-141 causes a more profound inhibitory effect on cell migration than that of each pre-miRNA separately (Liep et al., 2016); concurrent treatment with miRNA-145 and miRNA-143 mimics leads to a two-fold greater reduction in colorectal cancer cell viability (Su et al., 2014; Supplementary Table S1). In some esophageal squamous cell carcinoma cells, transfection of pre-miRNA-99a in combination with pre-miRNA-100 causes a 1.5-fold greater decrease in cell proliferation in contrast with monotherapy (Sun et al., 2013; Supplementary Table S1).

According to certain results, the application of miRNA mimic mixes exerts a therapeutic effect on several cellular functions. For instance, a combination of miRNA-99a and miRNA-497 mimics more effectively triggers apoptosis, as well as reduces hepatocellular carcinoma cell viability *in vitro*, as compared with each mimic alone (Cheng et al., 2017; Supplementary Table S1). The introduction of vectors driving the expression of both miRNA-34a and miRNA-497 provokes a 1.5-fold greater decrease in colony formation and cell viability than those driving expression of each mimic alone; however, neither an additive nor a synergistic influence on cell proliferation took place (Han et al., 2015; Supplementary Table S1). The viability and migratory ability of multiple myeloma cells were two-fold decreased following simultaneous treatment with pre-miRNA-137 and pre-miRNA-197 as compared with monotherapy (Yang et al., 2015; Supplementary Table S1). Joint restoration of miRNA-193a and miRNA-600 expression promotes a 1.5-fold greater reduction in colony formation and a up to 2.5-fold greater induction of apoptosis as compared with separate mimic treatment (Li et al., 2018; Supplementary Table S1). There exists only one example showing that the application of miRNA mimic cocktails can attain a synergistic effect on multiple events of tumorigenesis. Notably, restoration of tumor suppressor miRNA-34a and miRNA-126 levels in pancreatic adenocarcinoma cells has been shown to provoke the inhibition of cancer cell viability by up to 75%, which exceeds the effect of monotherapy by three-fold. At the same time, a considerable decrease in the migratory and invasive potentials (up to two-fold), as well as significant apoptosis induction, were established; approximately 30-50% of apoptotic cells are observed, which is four- and two-fold higher than the effects of miRNA-34a and miRNA-126 mimics alone, respectively (Feng et al., 2017; Figure 3, Table 1, and Supplementary Table S1).

The observed effectiveness of miRNA mimic combination therapy is sufficient for successful *in vivo* effects. Application of miRNA mimics *in vivo* has been demonstrated to be relatively potent in terms of tumor growth inhibition, and contributes to up to a two- and three-fold greater reduction in tumor node volume and weight, respectively, in comparison with monotherapy (Kasinski et al., 2014; Cheng et al., 2017; Supplementary Table S1). Moreover, in the case of simultaneous administration of miR-497 and miR-34a mimics, the *in vivo* effects of this mix are proven to be up to 4-fold more potent than each mimic alone (Han et al., 2015; Table 1 and Supplementary Table S1).

Results indicate the great potential of miRNA mimic application, and undoubtedly, the next step is the development of preclinical protocols for cooperative therapy of oncopathologies using combinations of synthetic miRNA mimics.

## Combination of miRna-Based Therapeutics and Chemotherapy to Reverse Drug Resistance

Chemotherapy remains one of the main methods for the treatment of oncopathologies. FDA-approved drugs, including temozolomide, gemcitabine, sunitinib, paclitaxel, and cetuximab, possess different mechanisms of antitumor action. Temozolomide, as an alkylating agent, promotes the initiation of apoptosis as a result of DNA damage during replication (Ohba and Hirose, 2016); sunitinib exhibits an anti-angiogenic effect by inhibiting tyrosine kinase receptors, in particular, the vascular endothelial growth factor receptor (Patel et al., 2016); gemcitabine, being a nucleotide analog bearing 2′-fluorine, blocks DNA synthesis (Song et al., 2016); cetuximab represents a monoclonal antibody that represses cell proliferation through the selective inhibition of the epidermal growth factor receptor (Li et al., 2015); and paclitaxel, belonging to the class of taxanes, exerts its cytostatic activity via suppression of the normal reorganization of microtubules during mitosis (Yardley, 2013). All these agents exhibit relatively high antitumor activity; however, the development of cancer cell resistance considerably reduces the efficacy of such therapy (Schlack et al., 2016). A plethora of evidence highlights the essential role of miRNAs in the development of resistant tumor cell phenotypes. A lack of sensitivity to several chemotherapeutics may be a consequence of the hyperfunction of oncogenic miRNAs such as miRNA-26a, miRNA-18a, miRNA-29b-1, miRNA-431, miRNA-4521, and miRNA-155, or may be the result of a significant decrease in the activity of tumor suppressor miRNAs, including miRNA-575, miRNA-642b, miRNA-4430, miRNA-203a, and miRNA-203b (Mikamori et al., 2017; Yamaguchi et al., 2017; Ge et al., 2018; Aako et al., 2019; Wang H. et al., 2019; Wang M. et al., 2019). Aberrant functions of miRNAs may impede the interactions between miRNAs and their mRNA targets, a few examples of which are miRNA-210-3p and the multidrug efflux transporter ABCC5 (also known as MRP5; Amponsah et al., 2017); miRNA-101 and DNA-dependent protein kinases (Hu et al., 2017); miRNA-125a and the pro-apoptotic protein A20 (Yao et al., 2016); miR-145 and the ribosomal protein S6 kinase p70S6K1 (Lin et al., 2016); miRNA-181b and the cylindromatosis protein CYDL (Takiuchi et al., 2013); miRNA-144-3p and the AT-rich interactive domain 1A protein ARD1A (Xiao et al., 2017); and miRNA-199a-5p and miRNA-375 with the PH domain and leucine-rich repeat protein phosphatase 1 PHLPP1 (Mussnich et al., 2015). An impaired interaction between miRNA and its mRNA target unescapably results in tumor cell resistance, since these targets are elements of key cellular signaling cascades such as NF-κβ, AKT, and JAK2/STAT3, which control apoptosis, cell cycle progression, DNA repair, RNA editing, and nucleotide synthesis (Wu et al., 2018; Chen et al., 2019). Based on these data, investigators have attempted to use combinations of miRNA-based therapeutics in conjunction with chemotherapeutics to overcome the development of resistance and to increase the efficacy of treatment. Two strategies for the combined application of miRNA-targeted agents and chemotherapeutic drugs have been developed. The first includes preliminary transfection of cancer cells with miRNA mimics followed by chemotherapy, and the second uses simultaneous treatment of miRNA-based therapeutics and chemotherapeutics (Figure 3).

Application of the first approach involves preliminary treatment with miRNA mimics that leads to the restoration of sensitivity of cells to chemotherapeutic agents and considerable enhancement of the effectiveness of subsequent chemotherapy. For instance, transfection with mimics of miRNA-429, miRNA-383, miRNA-101-3p, miRNA-195, miRNA-634, or miRNA-1294 results in a two- to 5-fold decrease in the values of EC_50_ and IC_50_ for gemcitabine, temozolomide, and paclitaxel (Fan et al., 2016; Yu et al., 2017; Chen et al., 2018; Tan et al., 2018; Jiang et al., 2019; Tu et al., 2019; Wang H. et al., 2019; Supplementary Table S1). A preparatory increase in cell sensitivity to gemcitabine by transfection with the synthetic tumor suppressor miRNA-205 has been shown to promote a two-fold decrease in the migratory potential of pancreatic cancer cells (Mittal et al., 2014; Supplementary Table S1). Moreover, 79% of cells are arrested in the G0/G1 phase of the cell cycle following combination treatment, which is 1.5-fold greater as compared with the use of the miRNA-205 mimic alone (Chaudhary et al., 2017; Supplementary Table S1). Another example is the precursory addition of miRNA-151a mimics to glioblastoma cells followed by temozolomide treatment, which contributes to the inhibition of colony formation promoting a three-fold decrease in the number of colonies *in vitro* (Zeng et al., 2018; Supplementary Table S1). In another case, transfection of metastatic renal carcinoma cells with miRNA-222 mimics followed by sunitinib treatment is 6-fold and two-fold more effective than monotherapy with miRNA mimic and sunitinib, respectively, in terms of angiogenesis inhibition (Khella et al., 2015; Supplementary Table S1).

As in the case of preliminary miRNA-based treatment, the strategy of simultaneous application of miRNA-based therapeutics and chemotherapies exerts high efficiency *in vitro*, providing an influence on the various events of tumorigenesis. Co-incubation of glioblastoma and colorectal cancer cells with sunitinib and an anti-miRNA-21 oligonucleotide or sunitinib and an miRNA-145 mimic attains up to a 75% decrease in cell viability, which is on average 1.5-fold more effective than each agent alone (Costa et al., 2013; Liu et al., 2015; Supplementary Table S1). Combination therapy with an miRNA-383 mimic and paclitaxel, an anti-miRNA-21 oligonucleotide and gemcitabine, and an miRNA-146 mimic and cetuximab promotes an average of two- to 2.5-fold more profound apoptosis induction in comparison with single-agent therapy (Huang et al., 2014; Li Y. et al., 2017; Jiang et al., 2019; Supplementary Table S1). In some exceptional cases, the percentage of apoptotic cells may be up to 6-fold higher than with mimic monotherapy (Dai et al., 2016; Table 1 and Supplementary Table S1). An approximate 3.5-fold effective reduction in the invasive or migratory potentials of osteosarcoma, lung or pancreatic cancer cells was observed for combinations of an miRNA-34a mimic and celecoxib, an miRNA-let-7b mimic and paclitaxel, and an miRNA-205 mimic and gemcitabine, respectively, as compared with single therapeutics (Mittal et al., 2014; Dai et al., 2016; Chen et al., 2017; Table 1 and Supplementary Table S1).

The efficiency of combination therapy with chemotherapeutics and miRNA mimics or anti-miRNA oligonucleotides has also been confirmed *in vivo.* Different combinations of chemotherapy, including gemcitabine, temozolomide, sunitinib, or paclitaxel, and miRNA-based therapeutics such as synthetic miRNA-205, miRNA-151à, miRNA-1291, and miRNA-let-7b mimics, or an anti-miRNA-21 oligonucleotide, have been shown to promote considerable inhibition of tumor growth, leading to a two- to four-fold retardation of tumor growth in comparison with monotherapy with each agent (Costa et al., 2015; Dai et al., 2016; Chaudhary et al., 2017; Li Y. et al., 2017; Mondal et al., 2017; Yu et al., 2017; Zeng et al., 2018; Tu et al., 2019; Table 1 and Supplementary Table S1). Moreover, in the case of concurrent miRNA-218 mimic and temozolomide administration, a 40-fold inhibition of tumor progression is achieved (Fan et al., 2015; Table 1). In addition, using combinations of antisense oligonucleotide targeted to miR-21 with gemcitabine achieves complete elimination of liver metastases as well as application of an miR-151a mimic and temozolomide significantly increases mouse survival rate (Li Y. et al., 2017; Zeng et al., 2018; Figure 3, Table 1, and Supplementary Table S1).

## Conclusion

All the described strategies based on the combined application of miRNA-based therapeutics, such as mixes of antisense oligonucleotides or miRNA mimics, as well as their combination with chemotherapeutics, exhibit high therapeutic efficacy. Although many publications evaluated neither the additive nor synergistic effects of the therapeutic mixes, in some, a significant enhancement of the therapeutic efficacy was demonstrated upon their application. In particular, an additive influence on the cell viability or invasion was observed for an Mtg AMO-21/155/17 (Lu et al., 2009) and an miRNA-101 mimic and gemcitabine (Fan et al., 2016) or for an miRNA-34a mimic with an miRNA-126 mimic or celecoxib, respectively (Chen et al., 2017; Feng et al., 2017; Supplementary Table S1). Moreover, miRNA-34a and miRNA-let-7b mimics and a miRNA-1291 prodrug with nab-paclitaxel-conjugated gemcitabine inhibit tumor growth *in vivo* in an additive manner (Kasinski et al., 2014; Tu et al., 2019; Supplementary Table S1). A very limited number of publications noted a synergistic influence of combinations on tumorigenesis (Table 1). For instance, mixes of anti-miRNA-221 and anti-miRNA-222 R8 conjugates, anti-miRNA-21 and anti-miRNA-10b ONs, miRNA-34a and miRNA-126 mimics, or an miRNA-634 mimic and temozolomide promote a synergistic manifold enhancement of apoptosis induction (Dong et al., 2012; Brognara et al., 2015; Feng et al., 2017; Tan et al., 2018), inhibition of colony formation (Zhang et al., 2015b; Tan et al., 2018), and a decrease in the invasive and migratory potential of cancer cells as compared with monotherapy (Dong et al., 2012; Kasinski et al., 2014; Chen et al., 2017; Table 1 and Supplementary Table S1). In addition, a number of combinations provide synergistic inhibition of tumor growth *ex vivo* and *in vivo*, such as miRNA-34a and miRNA-497 mimics, miRNA-205 and gemcitabine, and miRNA-let-7b and paclitaxel (Han et al., 2015; Dai et al., 2016; Chaudhary et al., 2017; Table 1 and Supplementary Table S1). In the most outstanding cases, a 40-fold retardation of tumor growth was attained using an miRNA-218 mimic and temozolomide (Fan et al., 2015), and complete elimination of metastases employing an anti-miRNA-21 ON and gemcitabine was achieved (Li Y. et al., 2017; Table 1 and Supplementary Table S1). These combinations may act as prototypes for therapeutic regimens against malignancies such as hepatocellular carcinoma, pancreatic cancer, and glioblastoma. All the analyzed data suggest that the basis of successful application of miRNA-based therapeutics lies in the choice of optimal miRNA targets that regulate key cellular pathways and form complicated networks of interactions with protein and mRNA targets in particular tumor cells.

The analyzed data demonstrate that miRNA-based therapeutics generate an entirely novel therapeutic paradigm in cancer treatment. It should be noted that miRNA-based therapeutics possess significantly less toxicity in contrast to existing drugs, in particular chemotherapeutics. For this reason, miRNA-based modalities may be applied as adjuvant agents to chemotherapy, allowing significant reductions in the dosage of antitumor drugs currently used in the clinic. Application of miRNA-based therapeutic mixes is, without question, an evolving area of antisense technology, and combinations of anti-miRNA ONs, mimics, and chemotherapeutics may represent highly efficient approaches to treating oncopathologies and other miRNA-associated diseases in the near future.

## Author Contributions

SM analyzed and published the data, and prepared the manuscript. OP revised and corrected the manuscript.

## Conflict of Interest Statement

The authors declare that the research was conducted in the absence of any commercial or financial relationships that could be construed as a potential conflict of interest.
