# Supplementary material for: Enhanced Inhibition of Tumorigenesis Using Combinations of miRNA-Targeted Therapeutics
Source: Front Pharmacol. 2019 May 16;10:488. doi: 10.3389/fphar.2019.00488 (PMC6531850; doi:10.3389/fphar.2019.00488)
Supplement: Supplementary file 1 [file Data_Sheet_1.docx]

Supplementary Material

**

**

**Figure S1. Structures of chemical modifications discussed in this review.**

**Table S1. In vitro and in vivo effectiveness of cancer cells treatment with mixes and single miRNA-based therapeutics**

| **Therapy** | **Inhibitors structure** | **Monotherapy effects** | **Combined therapy effects** | | **In vitro/**  **In vivo** | **Type of malignancy, cell line** | | **Reference** |
| --- | --- | --- | --- | --- | --- | --- | --- | --- |
| **Mixes of antisense oligonucleotides/multitarget sponges targeted to oncogenic miRNAs** | | | | | | | | |
| Anti-miRNA-17 and anti-miRNA-20a | LNA ONs | **Proliferation (%*****)**: 50% (anti-miRNA-17); 90% (anti-miRNA-20a); 90% (scramble control) | | **Proliferation (%)**: 30% | In vitro | | Lung cancer, ACC-LC-172 | (Matsubara et al., 2007) |
| Anti-miRNA-183, anti-miRNA-182 and anti-miRNA-96 | Commercial GeneChem (Shanghai, China) | **Proliferation (fold*):** 0.78 (anti-miRNA-183); 0.75 (anti-miRNA-182 and anti-miRNA-96);  **Colony formation (fold*):** 0.35 (anti-miRNA-183); 0.45 (anti-miRNA-182); 0.55 (anti-miRNA-96); 1.05 (scramble сontrol) | | **Proliferation (fold*):** 0.2; **Colony formation (fold*):** 0 | In vitro | | Colon cancer, HT-29 | (Zhang et al., 2015a) |
| Anti-miRNA-130a and anti-miRNA-495 | Commercial Qiagen (Netherlands) | **Angiogenesis (fold*):** Hemoglobin level (fold): 0.6 (anti-miRNA-495); 0.4 (anti-miRNA-130a) | | **Angiogenesis (fold*):** Hemoglobin level (fold): 0.3 | In vitro | | Gastric cancer, SNU5, SNU16, SNU484, MKN1, and MKN45 | (Lee et al., 2015) |
| Anti-miRNA-221 and anti-miRNA-222 | PNA conjugates with R8 peptide | **Late apoptosis (%): U251:** 2.13% (anti-miRNA-221); 2.14% (anti-miRNA-222); 1.54% (control); **U373:** 3.03% (anti-miRNA-221); 3.33% (anti-miRNA-222); 3.13% (control); **T98G:** 5.2% (anti-miRNA-221); 5.4% (anti-miRNA-222); 2.79% (control) | | **Late apoptosis (%):**  **U251:** 9.4%;  **U373:** 8.2%;  **T98G:** 14.4% | In vitro | | Glioma, U251, U373 and T98G | (Brognara et al., 2015) |
| Anti-miRNA-106b, anti-miRNA-93 and anti-miRNA-25 | Commercial GenePharma Co., Ltd, (Shanghai, China) | **Proliferation (amount of cells):**  10^5^ (anti-miRNA-106b, anti-miRNA-93 and anti-miRNA-25); 1.3×10^5^ (control);  **Migration (rate):** 50 (anti-miRNA-106b); 63(anti-miRNA-93); 82(anti-miRNA-25); 85(control); **Invasion (number of cells):** 40 ± 6 (anti-miRNA-106b); 45± 3 (anti-miRNA-93); 92± 5 (anti-miRNA-25); 103 ± 6 (control); **Apoptosis (%):** 10.6 ± 1.7 (anti-miRNA-106b); 8.9 ± 0.5% (anti-miRNA-93); 11.5 ± 1.5% (anti-miRNA-25);8.4± 1.1 (control) | | **Proliferation (Amount of cells):** 5×10^4^;  **Migration (rate):** 40; **Invasion (number of cells):** 24 ± 4;  **Apoptosis (%):** 16.1 ± 2.1% | In vitro | | Gastric cancer, MGC 803 | (Zhang R. et al., 2016) |
| Anti-miRNA-221 and anti-miRNA-222 | 2'OMe ONs | **Proliferation (fold*):** 0.64 (anti-miRNA-221 and anti-miRNA-222); 0.95(scramble control); **Cells in G1 phase (%):** 30.6% (anti-miRNA-221); 29.8% (anti-miRNA-222); 26.0% (scramble control); **Tumor volume (mm^3^):** 2500 mm^3^ (anti-miRNA-221 and anti-miRNA-222); 3100mm^3^ (control) | | **Proliferation (fold*):**0.42;  **Cells in G1 phase (%):** 46.2%;  **Tumor volume (mm^3^):** 1600 mm^3^ | In vitro, In vivo | | Glioblastoma, U251 | (Zhang et al., 2009) |
| Anti-miRNA-21 and anti-miRNA-10b | 2'OMe ONs | **Proliferation (%*):** 65±5.12% (anti-miR-10b); 75.2 ±9.00% (anti-miR-21); 95 ± 2.5% (scramble control);  **Cells in G1 phase (%):** 50 (anti-miR-10b); 57%(anti-miR-21); 35%(scramble control);  **Invasion (number of cells):** 200/field (anti-miR-10b); 170/field (anti-miR-21); 350/field (scramble control) | | **Proliferation (%*):** 52.80 ±10.18%;  **Cells in G1 phase (%):** 67%;  **Invasion (number of cells):** 50/field | In vitro | | Glioblastoma, U87MG | (Dong et al., 2012) |
| Multitargeted anti-miRNA-21/ miRNA-155/ miRNA-17 ON | DNA MTg-AMO | **Proliferation (%*):** 40% (anti-miR-155); 52% (anti-miR-21); 78% (anti-miR-17) | | **Proliferation (%*):** 18% MTg-AMO; 35%(mix of separate anti-miRNA ONs) | In vitro | | Breast cancer, MCF-7 | (Lu et al., 2009) |
| Multitarget sponge targeted to miRNA-155, miRNA-21, miRNA-221 and miRNA-222 | Endogenously expressed transcript (DNA) | **Proliferation (%*):** 0.92 (anti-miRNA-21 sponge); 0.85 (anti-miRNA-155 sponge); 0.8 (anti-miRNA-221 sponge) | | **Proliferation (%*):** 0.5 | In vitro | | Breast cancer, MDA-MB-436, MCF-7 | (Jung et al., 2015) |
| Multitarget sponge targeted to miRNA-17, miRNA-18a, miRNA-19, and miRNA-92 | Endogenously expressed transcript (DNA) | **Proliferation (%*):** 65% (anti-miR-18a sponge); 50% (anti-miR-19 sponge); 25% (anti-miR-17 and anti-miR-20 sponges); 20% (anti-miR-92 sponge); 90%(scramble control) | | **Proliferation (%*):** 10% (miRNA-17, miRNA-18a, miRNA-19, and miRNA-92 multitargeted sponge) | In vitro | | Hodgkin lymphoma, KM-H2 | (Kluiver et al., 2012) |
| **Therapy** | **Inhibitors structure** | **Monotherapy effects** | | **Combined therapy effects** | **In vitro/In vivo** | | **Cancer type (cell line)** | **Reference** |
| **Mixes of synthetic miRNA mimics to restore tumor suppressor miRNAs function** | | | | | | | | |
| Pre-miRNA-34a, pre-miRNA-15a and pre-miRNA-16 | Commercial Ambion (NY, USA) | **Cells in G1-G0 phase (%):** 20% (pre-miRNA-15a/-16); 42% (pre-miRNA-34a); 5% (control) | **Cells in G1-G0 phase (%):** 57% | | In vitro | Non-small cell lung cancer, A549, H2009, H1299 and H358 | | (Bandi and Vasella, 2011) |
| MiRNA-34a and miRNA-let-7b mimics | Commercial Ambion (NY, USA) | **Proliferation (%*):** 75% (miRNA-Let-7b); 60% (miRNA-34a);  **Invasion (number of cells):**80/ field (miRNA-34a); 53/field (miRNA-let-7b); 55/field (control);  **Tumor area (fold*):** 0.75 (miRNA-let-7b); 0.7(miRNA-34a) | **Proliferation (%*):** 48%;  **Invasion (number of cells):** 10/per field;  **Tumor area (fold*):** 0.45 | | In vitro, in vivo | Lung cancer, A549, H441 and H23 | | (Kasinski et al., 2014) |
| Pre-miRNA-141 and pre-miRNA-145 | Commercial Life Technologies (USA), Ambion (Darmstadt, Germany) | **Migration (fold*):** 0.84 (Pre-miRNA-141 and pre-miRNA-145) | **Migration (fold*):**  0.67 | | In vitro | Renal cell Carcinoma, 786-O and ACHN | | (Liep et al., 2016) |
| Pre-miRNA-143 and pre-miRNA-145 | Commercial GenePharma Co., Ltd, (Shanghai, China) | **Proliferation (fold*):** 0.65 (pre-miRNA-143); 0.6 (pre-miRNA-145) | **Proliferation (fold*):** 0.35 | | In vitro | Colorectal cancer, Caco2, HT29 and SW480 | | (Su et al., 2014) |
| Pre-miRNA-99a and pre-miRNA-100 | Commercial | **Proliferation (fold*):** 0.6 (pre-miRNA-99a); 0.75 (pre-miRNA-100); 1.25 (scramble control) | **Proliferation (fold*):** 0.5 | | In vitro | Esophageal squamous cell carcinoma, EC9706 | | (Sun et al., 2013) |
| MiRNA-99a and miRNA-497 mimics | Commercial Ribo (Guangzhou, China) | **Proliferation (fold*):** 0.8 (miRNA-99a mimic); 0.77 (miRNA-497 mimic); 1.1 (scramble control); **Apoptosis (%):** 19.22% (miRNA-99a); 26.28% (miRNA-497); 4.87% (control);  **Tumor volume (mm^3^):** 750 mm^3^ (miRNA-497); 600 mm^3^ (miRNA-99a); 1300 (control);  **Tumor weight (g):** 0.8 g (miRNA-497); 0.6 g (miRNA-99a); 1.33 g (control) | **Proliferation (fold*):** 0.6;  **Apoptosis (%):** 28.27%;  **Tumor volume (mm^3^):** 250 mm^3^;  **Tumor weight (g):** 0.25 g | | In vitro, in vivo | Hepatocellular carcinoma, Hep2G and Hep3B | | (Cheng et al., 2017) |
| MiRNA-497 and miRNA-34a mimics | CommercialGenePharma (Shanghai, China) | **Proliferation (fold*):** 0.43 (miRNA-34a mimic); 0.45 (miRNA-497 mimic); **Colony formation (%*):** 55% (miRNA-34a mimic); 60% (miRNA-497 mimic); **Tumor volume (mm^3^):** 200 mm^3^ (miRNA-34a); 180 mm^3^ (miRNA-497); 450 mm^3^ (control); **Tumor weight (g):** 0.55 g (miRNA-34a); 0.45g (miRNA-497); 0.75g (control) | **Proliferation (fold*):** 0.28; **Colony formation (%*):**40%; **Tumor volume (mm^3^):** 50 mm^3^;  **Tumor weight (g):**0.2 g | | In vitro, ex vivo | Lung cancer, A549, H1299, H460, H446, and QG56 | | (Han et al., 2015) |
| Pre-miRNA-137 and pre-miRNA-197 | Commercial GeneCopeia (USA) | **Proliferation (fold*):** 0.6(miRNA-137); 0.5(miRNA-197) **Migration (fold):** 0.5(miRNA-137); 0.55(miRNA-197) **Colony formation (number of colonies):** 45(miRNA-137); 42(miRNA-197); 80(scramble control) | **Proliferation (fold*):** 0.35; **Migration (fold):** 0.3;  **Colony formation (number of colonies):** 32 | | In vitro | Multiple myeloma, MM1.S, MM1.R, OCI-My5, NCI-H929 and U266 | | (Yang et al., 2015) |
| MiRNA-193a and miRNA-600 mimics | Endogenously expressed from lentivirus vector | **Colony formation (number of colonies):** 90(miRNA-193a mimic); 100 (miRNA-600 mimic); 160 (scramble control); **Apoptosis (%):** 23%(miRNA-193a); 17%(miRNA-600); 7%(control) | **Colony formation (number of colonies):** 50;  **Apoptosis (%):** 35% | | In vitro, in vivo | Acute myeloid leukemia, K562 and THP1 | | (Li et al., 2018) |
| MiRNA-126 and miRNA-34a mimics | Endogenously expressed from adenovirus vector | **Proliferation (%*):** 80%(miRNA-126 and miRNA-34a mimics); 83%(scramble control); **Migration (number of cells): Panc-1:** 90(miRNA-126); 80(miRNA-34a); 140(control);  **Invasion (number of cells): Panc-1:** 75(miRNA-126 mimic); 70 (miRNA-34a mimic); 125 (control);  **Apoptosis (%): Panc1:** 13% (miRNA-126); 25%(miRNA-34a); 8%(control); **Capa-2:** 5%(miRNA-126); 16%(miRNA-34a); 8%(control);  **Tumor volume (mm^3^):** 1600 mm^3^ (miRNA-126); 1450 mm^3^ (miRNA-34a); 2250 mm^3^ (control); **Tumor weight (g):** 2 g (miRNA-126); 1.6 g (miRNA-34a); 2.7 g (control) | **Proliferation (%*):** 25%;  **Migration (number of cells): Panc-1:** 50;  **Invasion (number of cells): Panc-1:** 30;  **Apoptosis (%):**  **Panc1:**50%;  **Capa-2:** 30%;  **Tumor volume (mm^3^):**  1000 mm^3^;  **Tumor weight (g):** 1 g | | In vitro, in vivo | Pancreatic adenocarcinoma, Panc-1 and SW1990 | | (Feng et al., 2017) |
| **Combinations of chemotherapeutic drugs and miRNA-based therapeutics** | | | | | | | | |
| Pre-miRNA-429 and gemcitabine (Gem) | Commercial Ribobio (Guangzhou, China) | **IC50 Gem (µM)**: 215 µM; **Tumor volume (mm^3^):** 620 mm^3^ (Gem) | **IC50 Gem (µM):** 115 µM; **Tumor volume (mm^3^):** 300mm^3^ | | In vitro, in vivo | Pancreatic cancer, SW1990 | | (Yu et al, 2017) |
| MiRNA-101 mimic and gemcitabine | Commercial Quiagen (Hilden, Germany): | **Proliferation (fold*): PANC-1:**  0.35 (Gem); 0.39 (miRNA-101 mimic); **Bx:** 0.84 (Gem); 1.0 (miRNA-101 mimic); **AsPC-1:** 0.53 (Gem or miRNA-101 mimic) | **Proliferation (fold*): PANC-1:** 0.19; **Bx:** 0.61; **AsPC-1:** 0.38 | | In vitro | Pancreatic ductal adenocarcinoma, PANC-1, AsPC-1, MIA-PaCa2, AsanPaCa and BxPC-3 | | (Fan et al., 2016) |
| MiRNA-634 and temozolomide (TMZ) | Commercial GeneCopoecia, (Guangzhou, China) | **IC50 TMZ (µM): U87:** 240 µM; **U251:** 260 µM; **Proliferation (%*): U87:** 25% (TMZ); **U251:** 45% (TMZ); **Apoptosis (%):** 15%(miRNA-634); 16%(TMZ); 7% (control); **Colony formation (%*):** 55% (miRNA-634); 57%(TMZ) | **IC50 TMZ (µM): U87:** 120 µM; **U251:** 100 µM; **Proliferation (%*): U87:** 8%; **U251:** 10%;  **Apoptosis (%):** 27%;  **Colony formation (%*):** 18% | | In vitro | Glioma, U251 and U87 | | (Tan et al., 2018) |
| MiRNA-1294 mimic and temozolomide | Commercial GenePharma (Shanghai, China) | **IC50 TMZ (µM): U87:** >400 µM; **U251:** >400 µM;  **Proliferation (%*): U87 and U251:** 60% (TMZ) | **IC50 TMZ (µM): U87:** 150 µM; **U251:** 200 µM;  **Proliferation (%*): U87:** 20%; **U251:** 35% | | In vitro | Glioma, U87, U251, LN229 and A172 | | (Chen et al., 2018) |
| MiRNA-383 mimic and paclitaxel | Commercial GenePharma (Shanghai, China) | **IC50 Paclitaxel (µM): OVCAR-3:** 3.0 µM; **A2780:** 1.5 µM;  **Apoptosis (%): A2780:** 13% (paclitaxel); 4% (control); **OVCAR-3:** 8% (paclitaxel); 3% (control) | **IC50 Paclitaxel (µM): OVCAR-3:** 1.8 µM; **A2780:** 0.3 µM;  **Apoptosis (%): A2780:** 27%; **OVCAR-3:** 22% | | In vitro | Cervical cancer, A2780 and OVCAR-3 | | (Jiang et al., 2019) |
| MiRNA-1291 prodrug and gemcitabine and nab-paclitaxel | Endogenously expressed from plasmid | **EC50 GEM/nP (nM): PANC-1:** 155±33 nM; **AsPC-1 cells:** 40.4±1.8 nM;  **Tumor weight (mg):** 200mg (miRNA-1291 prodrug); 240 mg(gem/nP); 400 mg (control) | **EC50 GEM/nP (nM): PANC-1:** 52.3±20.3 nM; **AsPC-1:** 14.6±5.5 nM;  **Tumor weight (mg):** 100 mg | | In vitro, in vivo | Pancreatic cancer, AsPC-1, PANC-1, and HEK293 | | (Tu et al., 2019) |
| MiRNA-205 mimic and gemcitabine | Endogenously expressed from lentivirus vector | **MIA PaCa-2R cells in G0/G1 phase:** 52.62 ±0.002% (miRNA-205 mimic); 43.52 ±0.06% (control);  **Tumor volume (mm^3^):** 172.85 ± 17 mm^3^ (gem); 298.46±54 mm^3^ (miRNA-205 mimic); 475±100 mm^3^ (control) | **MIA PaCa-2R cells in G0/G1 phase:** 78.54 ± 0.01%;  **Tumor volume (mm^3^):**  77.83 ± 21 mm^3^ | | In vitro, in vivo | Pancreatic cancer, MIA PaCa-2, HPAF-II, BXPC-3, HPDE and MIA PaCa-2R | | (Chaudhary et al., 2017) |
| MiRNA-151a mimic and temozolomide | Commercial miRNA-151a expressing vector Genechem, (Shanghai, China) | **Colony formation (number of colonies):** 330 (miRNA-151a mimic); 300 (TMZ); 380(control);  **IC50 TMZ:** 400 µM; **Tumor volume (mm^3^):** 1.0 cm^3^ (miRNA-151a mimic); 0.8 cm^3^ (TMZ); 1.1 cm^3^ (control); **Survival (days):** 87 days (TMZ); 75 days (miRNA-151a mimic); 65 (control) | **Colony formation (number of colonies):** 100;  **IC50 TMZ:** 150 µM;  **Tumor volume (mm^3^):** 0.3 mm^3^;  **Survival (days):** 150 days | | In vitro, in vivo | Glioblastoma, U251, T98G, LN229 and A172 | | (Zeng et al., 2018) |
| MiRNA-222 mimic and sunitinib | Commercial Applied Biosystems (USA) | **Angiogenesis (total tube length):** 3000 pχ(miRNA-222 mimic); 1000 pχ (sunitinib); 4800 pχ (control) | **Angiogenesis (total tube length):** 500 pχ | | In vitro | Kidney cancer, ACHN | | (Khella et al., 2015) |
| Anti-miRNA-21 oligonucleotide and sunitinib | Commercial LNA asON Exiqon (Vedbaek, Denmark) | **Caspases 3/7 activity (fold*):** 1.6 (sunitinib); 4.5 (anti-miRNA-21 ON); 2.0 (scramble control); **Proliferation (%*):** 78.2±10.8 (sunitinib or anti-miRNA-21) | **Caspases 3/7 activity (fold*):** 8.15;  **Proliferation (%*):** 56.52±14.48 | | In vitro | Glioblastoma, U87 | | (Costa et al., 2013) |
| MiRNA-145 mimic and sunitinib | Commercial Dharmacon (Lafayette, CO) | **Proliferation (%*):** 42.8±6.2% (sunitinib); 50±2 % (miRNA-145 mimic);  **Apoptosis (%):** 18%(miRNA-145 mimic); 15% (sunitinib); 5%(control);  **Cells in G0/G1 phase (%):** 65% (sunitinib); 78%(miRNA-145 mimic); 55% (control) | **Proliferation (%*):** 28.5±5.1%;  **Apoptosis (%):** 36%;  **Cells in G0/G1 phase (%):** 90% | | In vitro | Glioblastoma, U87 | | (Liu et al., 2015) |
| Pre-miRNA-133b and cetuximab | Vector expressing pre-miRNA-133b | **Invasion (fold*):** 0.3(cetuximab); 0.27 (pre-miRNA-133b); 0.43 (scramble control) | **Invasion (fold*):** 0.15 | | In vitro | Colorectal cancer, HT-29, SW480, SW620, Caco-2 and HCT-116 | | (Zhou et al., 2015) |
| Anti-miRNA-21 and gemcitabine | Commercial GenePharma (Shanghai, China) | **IC_50_GEM (µM): PANC‐1:** 38.92 μM; **Mia PaCa‐2:** 38.29 μM;  **Apoptosis (%): PANC‐1:** 15% (gem); 25% (anti-miRNA-21); 10% (control); **Mia PaCa‐2:** 15% (gem); 25% (anti-miRNA-21); 9% (control); **Proliferation (Mia PaCa‐2) (%*):** 65% (gem); 50% (anti-miRNA-21); 105% (scramble control);  **Tumor weight (g):** 0.5 g (anti-miRNA-21); 0.65 g (gem); 0.9 g (control);  **Tumor volume (mm^3^):** 300 mm^3^ (anti-miRNA-21); 560 mm^3^ (gem); 800 mm^3^ (control);  **Liver metastases (number per field):**  1.5/field (anti-miRNA-21); 3.8/field (gem); 3.3/field | **IC_50_GEM (µM): PANC‐1:** 22.38;  **Mia PaCa‐2:** 6.65 μM;  **Apoptosis (%): PANC‐1:** 50%; **Mia PaCa‐2:** 45%;  **Proliferation (Mia PaCa‐2) (%*):**30%;  **Tumor weight (g):** 0.11 ± 0.01 g;  **Tumor volume (mm^3^):** 145.00 ± 7.25 mm^3^;  **Liver metastases (number per field):** Complete elimination of liver metastases | | In vitro, in vivo | Pancreatic cancer, PANC‐1 and MIA PaCa‐2 | | (Li Y. et al., 2017) |
| MiRNA-146a mimic and cetuximab | CommercialAmbion (Life Technologies Grand Island, NY, USA) | **Proliferation (HepG2) (%*):** 45% (miRNA-146a mimic or cetuximab); **Caspase 3/7 activity (fold*):** 1.3 (miRNA-146a mimic); 4 (cetuximab) | **Proliferation (HepG2) (%*):** 30%;  **Caspase 3/7 activity (fold*):** 5.5 | | In vitro | Hepatocellular carcinoma, HepG2, HepB3 and SNU449 | | (Huang et al., 2014) |
| MiRNA let-7b mimic and paclitaxel | Commercial Bioneer (Alameda, CA) | **Apoptosis (%):** 11.2% (paclitaxel); 4.4% (let-7b mimic); 2% (control);  **Invasion (number of cells):** 150/field (paclitaxel); 180/field (let-7b mimic); 280/field (control);  **Tumor volume (mm^3^):** 450 mm^3^ (paclitaxel); 650mm^3^ (let-7b mimic); 850 mm^3^ (control) | **Apoptosis (%):** 27.7%;  **Invasion (number of cells):** 50/field;  **Tumor volume (mm^3^):** 150 mm^3^ | | In vitro, in vivo | Non-small cell lung cancer, A549 | | (Dai et al., 2016) |
| MiRNA-34a mimic and celecoxib | Commercial | **Proliferation (%*):** 62.9% (miRNA-34a mimic); 53.1% (celecoxib);  **Invasion (number of cells):** 25.2 (miRNA-34a mimic); 21.3 (celecoxib); 30 (control);  **Migration (distance):** 135µm (celecoxib); 80 µm (miRNA-34a mimic); 180µm (control) | **Proliferation (%*):** 44.9%;  **Invasion (number of cells):** 12.6;  **Migration (distance):** 35 µm | | In vitro | Osteosarcoma, MG63 | | (Chen et al., 2017) |
| MiRNA-205 mimic and gemcitabine | Commercial Life Technologies (Carlsbad, CA) | **Invasion (%*):** 60% (miRNA-205 mimic); 145% (gem);  **Migration (%*):** 75%(miRNA-205 mimic); 110% (gem);  **Proliferation (%*): MIA PaCa-2^R^:** 90% (gem); **CAPAN-1^R^:** 85% (gem); **Tumor weight (g):** 0.53 g (gem); 0.93 g (Control) | **Invasion (%*):** 55%;  **Migration (%*):** 30%;  **Proliferation (%*): MIA PaCa-2^R^:** 42%; **CAPAN-1^R^:** 46%;  **Tumor weight (g):** 0.14 g | | In vitro, in vivo | Pancreatic cancer, M IA PaCa-2^R^ and CAPAN-1^R^ | | (Mittal et al., 2014) |
| Anti-miRNA-21 and sunitinib | Commercial LNA anti-miRNA-21 ON Exiqon (Vedbaek, Denmark) | **Tumor volume (mm^3^):** 90.9 ± 18.2 mm^3^ (sunitinib); 98.2 ± 43.8 mm^3^ (control) | **Tumor volume (mm^3^):** 53.7 ± 43.7 mm^3^ | | In vivo | Glioblastoma, GL261 | | (Costa et al., 2015) |
| MiRNA-205 mimic and gemcitabine | Commercial Life Technologies (Carlsbad, CA) | **Tumor weight (g):** 0.75 (gem); 0.9 g (control); **Tumor volume (mm^3^):** 800 mm^3^ (gem); 900 mm^3^ (control) | **Tumor weight (g):** 0.2 g; **Tumor volume (mm^3^):** 100 mm^3^ | | In vivo | Pancreatic ductal adenocarcinoma | | (Mondal et al., 2017) |
| MiRNA-218 mimic and temozolomide | Commercial | **Tumor weight (g):** 0.2 g (miRNA-218 mimic); 0.05 g (TMZ); 0.42 g (control) | **Tumor weight (g):** 0.01 g | | In vivo | Glioblastoma, U87MG | | (Fan et al., 2015) |

*The effect observed for control intact cells is equal to 100% or 1.0.
